# Living-donor parathyroid allotransplantation for therapy-refractory postsurgical persistent hypoparathyroidism in a nontransplant recipient – three year results: a case report

**DOI:** 10.1186/s12893-016-0165-y

**Published:** 2016-08-03

**Authors:** Ayman Agha, Marcus Nils Scherer, Christian Moser, Thomas Karrasch, Christiane Girlich, Fabian Eder, Ernst-Michael Jung, Hans Juergen Schlitt, Andreas Schaeffler

**Affiliations:** 1 Department of Surgery, University Hospital Regensburg, Franz-Josef-Strauss-Allee 11, D-93053 Regensburg, Germany; 2 Department of Internal Medicine I, University Hospital Regensburg, D-93053 Regensburg, Germany; 3 Medical Clinic and Policlinic III, University Hospital Giessen, D-35392 Giessen, Germany; 4 Department of Pathology, University Hospital Regensburg, D-93053 Regensburg, Germany; 5 Department of Radiology, University Hospital Regensburg, D-93053 Regensburg, Germany

**Keywords:** Hypoparathyroidism, Hypocalcemia, Living-donor parathyroid allotransplantation, Immunosuppression

## Abstract

**Background:**

Therapy-refractory persistent hypoparathyroidism after extensive neck surgery is a rare but severe complication. Parathyroid allotransplantation may represent a definitive treatment option.

**Case presentation:**

A 32-year old female was referred to our hospital with intractable persistent hypocalcemia after neck surgery for papillary thyroid cancer. Despite optimal medical treatment including calcium and vitamin D supplementation and even hormonal replacement therapy hypocalcemic symptoms failed to improve. The quality of life was considered very low. In light of the unsuccessful medical therapy and the young age of the patient parathyroid allotransplantation seemed an attractive treatment option to restore normal calcium homeostasis despite of the need for immunosuppressive therapy after the procedure. Therefore, we performed living-donor allotransplantation of two healthy parathyroid glands to the recipient’s left forearm. The surgical intervention was successful. Neither the donor nor the recipient showed any complications. In the postoperative course clinical symptoms of hypocalcemia significantly improved whereas serum calcium and parathyroid hormone (PTH) levels progressively increased into the normal range. Former intense replacement therapy could be discontinued completely in a stepwise fashion. To date, nearly three years after transplantation, the patient remains asymptomatic with normal serum levels of calcium and PTH.

**Conclusion:**

Successful living-donor parathyroid allotransplantation for postsurgical hypoparathyroidism represents an innovative therapeutic strategy that could provide the definitive treatment in those patients in which the disease is therapy-refractory. The procedure can be justified even in nontransplant recipients. Retrieval of parathyroid glands from healthy donors is feasible and safe.

## Background

Postsurgical hypoparathyroidism with symptomatic hypocalcemia after neck surgery is a common but usually transient complication. However, if hypocalcemia persists for more than six months even with medical replacement therapy and cryopreserved autologous parathyroid tissue is not available, permanent hypoparathyroidism can lead to dependence on high doses of calcium and vitamin D supplementation [1–4]. Chronic hypocalcemia is significantly associated with longterm risks of multiorgan calcinosis and renal failure. Symptoms related to hypocalcemia can even be life-threatening (e.g. laryngeal spasm, refractory heart failure). Importantly, in rare cases hypocalcemia cannot be controlled despite of aggressive oral supplementation of calcium and vitamin D and frequent additional intravenous (i.v.) administration of calcium may be required seriously affecting the quality of life [5]. Recently, novel synthetic PTH-replacement therapies have been approved showing promising results for patients who do not respond well to treatment with calcium and active forms of vitamin D alone. However, long-term hormonal treatment may be limited because of the potential risk of bone cancer [6, 7].

Allotransplantation of parathyroid cells could be an ideal therapeutic option to establish physiologic treatment for patients with persistent hypoparathyroidism. Successful allotransplantation of parathyroid tissue from deceased and living donors has been described sporadically in recent years. However, most of these procedures were performed in kidney transplant recipients with ongoing immunosuppression [8–11].

We present - to the best of our knowledge – the first successful case of living-related allotransplantation of parathyroid glands obtained from a healthy donor in a nontransplant patient suffering from therapy-refractory postsurgical hypoparathyroidism.

## Case presentation

**Fig. 1:**
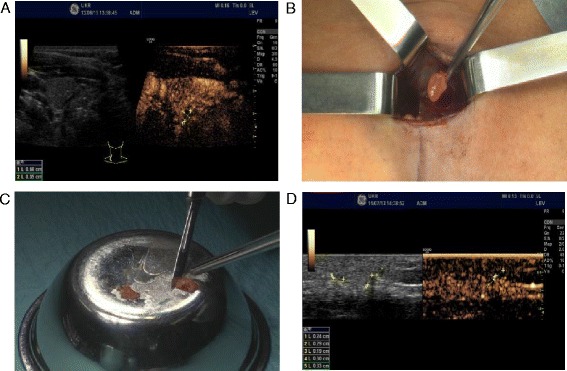
Living-donor allotransplantation of parathyroid glands. **a** Donor: preoperative localization of the parathyroid glands by CEUS **b** Donor: retrieval of the parathyroid allograft **c** Parathyroid glands were cut into approximately 20 tissue particles of 1–2 mm size **d** Recipient: postsurgical examination of the vascularization of transplanted parathyroid tissue by CEUS 10 days posttransplant

Our patient is a 32-year old white female patient who underwent surgery for multinodular goiter in 2006. During surgery, a papillary thyroid carcinoma had been detected (final histology: pT2, pN0, R0). Therefore, extensive neck dissection with total thyroidectomy was necessary leading to removal of all four parathyroid glands. Severe chronic postsurgical hypoparathyroidism developed rapidly. Despite optimal medical treatment with high doses of oral calcium (3 g/day), cholecalciferol (7000 IU/day) and calcitriol (2.0–3.75 μg/day) her calcium level remained low (6.25 mg/dl; normal 8.6–10.6 mg/dl) causing intolerable clinical hypocalcemic symptoms including tetany and paresthesias. Moreover, the patient frequently required hospitalization for i.v. calcium administration. Five years after thyroid surgery additional hormonal replacement therapy with parathyroid hormone PTH (1–84) was started (subcutaneous injection: 100 μg/day). However, the patient’s symptoms improved transiently for only three months. Altogether the quality of life was considered very low. Thus, allotransplantation of parathyroid glands for (definitive) treatment of persistent symptomatic and refractory hypocalcemia seemed an attractive therapeutic option. Despite of the potential risks associated with lifelong immunosuppressive therapy it was the distinct will of the patient to undergo the allotransplantation procedure. The patient’s 31-year old healthy brother expressed interest to be evaluated as a potential donor. He was ABO compatible to the recipient with no history of any relevant diseases and considered a suitable donor. Importantly, preoperative calcium and PTH-levels were within the normal range (Table 1). By using contrast-enhanced ultrasonography (CEUS) [12], three parathyroid glands (one left superior, one left inferior, one right inferior) could exactly be localized preoperatively (Fig. 1a). Importantly, the Hospital Living-Donation Committee as well as the Hospital Ethics Committee approved the planned operative procedure. After removing the two left parathyroid glands from the donor through an unilateral horizontal anterior neck incision (length 2.5 cm; Fig. 1b) and histological confirmation by frozen section the parathyroid tissue was fragmented into approximately 20 small particles (Fig. 1c) and implanted in the recipient’s left forearm brachioradialis muscle. Based on the the immunosuppressive therapy regimen for kidney transplantation, immunosuppression was initiated during surgery before implantation of parathyroid tissue by prednisolone i.v. (500 mg) and a perioperative single dose of basiliximab (20 mg). Additionally, oral application of tacrolimus was started six hours after surgery. Tacrolimus trough levels were maintained in the 10–12 ng/ml range. On day five after surgery, the patient received a second dose of basiliximab i.v. (20 mg). Prednisolone was reduced in a stepwise fashion to a maintenance dose of 2.5 mg per day after six months. Hormonal replacement therapy with PTH (1–84) was stopped after transplantation. Neither the donor nor the recipient experienced any surgical complications. The donor was discharged from our hospital on the second postoperative day and routine checks of calcium and PTH-levels showed normal values after surgery (Table 1). In the postoperative course of the recipient increasing PTH levels were detectable and thereafter remained within the normal range with a serum total PTH of 21 pg/ml (normal range: 15–65 pg/ml) nearly three years after transplantation (Table 2). Postoperative Casanova-Tests (venous blood samples for PTH measurement after inflation of an armlet on the graft-bearing arm) clearly verified the graft-bearing forearm as the source of increasing endogenous PTH levels after transplantation. Moreover, CEUS ten days posttransplant revealed vascularization of the allograft (Fig. 1d). The calcium levels of the recipient also returned into the normal range after parathyroid allotransplantation (Table 2). Consequently the former replacement therapy (calcium and vitamin D) could be discontinued stepwise completely and was stopped on day 16 after the transplantation. Six months posttransplant another Casanova-Test verified the functioning graft (PTH-level before and after compression of the graft-bearing forearm: 41 pg/ml and 5.9 pg/ml respectively). The patient remained free of hypocalcemic symptoms to the present day after a follow-up of 35 months so far.

**Table 1:** Serum calcium levels of the donor

			Months after transplant		
	Preoperative	Postoperative	3	6	12
Calcium [mg/dl]	9.2	8.8	9.2	8.4	8.8

No calcium or vitamin D supplementation; PTH within the normal range

**Table 2:** PTH values and serum calcium levels posttransplant (recipient)

	Months after transplant							
	2	3	8	10	19	27	30	35
PTH [pg/ml]	22	21	24	35	17	18	17.5	21
Calcium [mg/dl]	9.6	9.6	9.2	9.6	8.4	9.2	9.6	9.2

Normal range: PTH: 15–65 pg/ml; serum calcium: 8.6–10.6 mg/dl

## Discussion

Surgery for thyroid or parathyroid disease is the leading cause of hypoparathyroidism due to damage or (accidental) removal of parathyroid glands [2, 3, 13, 14]. In most cases resulting hypocalcemia and corresponding hypocalcemic symptoms can easily be controlled by oral calcium and vitamin D supplementation. However, in less frequent cases (0,4 – 33 %) the usually transitory postsurgical hypoparathyroidism persists for more than six months causing serious metabolic disorders with long-term risks of multiorgan calcinosis and renal failure [3]. Moreover, acute fall in serum calcium levels can even cause life-threatenig symptoms. Affected patients may frequently require i.v. calcium administration in addition to high doses of oral calcium and vitamin D supplements. Intractable hypocalcemic symptoms and repeated hospitalizations significantly reduce the quality of life in these patients [5]. Recently, novel synthetic PTH replacement therapies became available as a promising treatment option for patients who do not respond well to calcium and vitamin D supplementation alone. PTH (1–84) (Natpara; whole molecule) and PTH (1–34) (Forteo; active amino terminal portion) are injectable formulations (subcutaneous injection) [6, 7]. However, animal studies in rats showed that prolonged treatment with PTH (1–84) and PTH (1–34) is associated with the development of bone tumors (osteosarcoma) [15]. Although to date this response has not been observed in humans long-term therapy may be limited [16, 17]. Moreover, PTH (1–34) was approved by the Food and Drug Administration (FDA) only for therapy of osteoporosis. Therefore, treatment for hypoparathyroidism requires off label use. High monthly costs and an adequate patient compliance (inconvenient daily injections) are further aspects that have to be taken into account before starting a hormonal replacement therapy with either PTH (1–84) or PTH (1–34) [18].

The most physiological and potentially definitive therapy for persistent hypoparathyroidism would be a restoration of parathyroid tissue in order to re-establish calcium homeostasis. In this context parathyroid tissue allotransplantation from deceased or living donors has been performed sporadically over the past decades [8–11, 18]. Importantly, to avoid rejection of the graft lifelong immunosuppressive therapy of the recipient is necessary and therefore adverse effects of immunosuppression (e.g. impaired kidney function, opportunist infections, cancer) have to be carefully considered. However, searching the literature several authors reported on alternative experimental strategies to restore parathyroid tissue without the need for immunosuppressive therapy, e.g. by using cultured cells [8, 19], cryopreserved tissue [20, 21] or magnetic microspheres depletion of major histocompatibility complex (MHC) of cells from parathyroid adenoma [8]. Microencapsulation of parathyroid tissue constitutes another innovative concept aiming to exclude parathyroid cells from a host-versus-graft reaction. While the transplants are isolated from the immune system nutrients and hormones may pass the semipermeable membrane of the microcapsule. Initial results were encouraging showing normal PTH levels from the third week after transplantation [22] or graft survival up to 8 months [23]. However, Bohrer et al. recently could demonstrate that the promising results of microencapsulation of allogeneic parathyroid tissue are not due to the protection of the graft from the immune system, but rather derive from delaying immunization of the host [24]. Therefore, long-term effectiveness of this method may be limited.

The first successful parathyroid gland allotransplantation was published in 1973 by Growth et al. in a recipient who developed severe hypocalcemia after kidney transplantation [10]. The donor was a patient with parathyroid hyperplasia. As the recipient was already under immunosuppression and the donor needed surgical excision of parathyroid adenoma living donor allotransplantation seemed an attractive and justified therapy. The parathyroid graft survived 21 months. Since then several authors have described this procedure achieving various results concerning graft function [9, 25, 26]. Interestingly, Alfrey et al. published a case of a renal transplant patient with normocalcemia even 13 years after additional successful parathyroid allotransplantation [27]. The first report of a healthy patient who donated two parathyroid glands was published in 1975 by Wells et al. [28]. The recipient was a renal failure patient requiring total parathyroidectomy after kidney transplantation resulting in hypoparathyroidism. The father of the patient who previously had been his renal transplant donor additionally donated two healthy parathyroid glands resulting in successful treatment of hypocalcemia and associated symptoms of the recipient. The parathyroid grafts showed a good function for more than one year. Another similar report of a healthy patient donating one parathyroid gland to a family member who previously had undergone kidney transplantation shows a good functionig graft two years after parathyroid allotransplantation (normal PTH levels, oral calcium supplementation 1 g/day) [29]. Only recently, Garcia-Roca published the first successful case of simultaneous living donor kidney and pararthyroid allotransplantation (healthy family member donated kidney and one parathyroid gland) [30]. Nine months after transplantation the recipient remains asymptomatic with normal renal function and PTH levels (oral calcium supplementation 2 g/day). The authors conclude that parathyroid allograft transplantation can be justified in recipients who receive immunosuppressive therapy for another organ. However, for successful and potentially definitive treatment of debilitating hypocalcemia that is otherwise not controllable parathyroid gland allotransplantion might as well be a reasonable option for patients without immunosuppression because of previous transplantation of another organ. In this context Hermosillo-Sandoval et al. recently performed five parathyroid allografts in patients with iatrogenic hypoparathyroidism [31]. The living donors were patients with primary or secondary hyperparathyroidism requiring surgical excision. Four of the grafts are functioning two years after allotransplantation but calcium supplementation at a lower dose than before the procedure is necessary. No adverse effects of immunosuppression have been observed. In our case study the recipient also suffered from iatrogenic (postsurgical) hypoparathyroidism. The patient was otherwise healthy not receiving immunosuppressive therapy due to previous transplantation of another organ. Nevertheless parathyroid allotransplantation seemed a very attractive therapeutic option in light of the unsuccessful medical treatment including hormonal replacement therapy. To date the graft shows excellent function without the need for additional calcium supplementation. Furthermore, so far no adverse effects of the immunosuppressive drugs occurred and the patient significantly gained in quality of life. Therefore, in our opinion parathyroid gland allotransplantation can be justified also in nontransplant recipients. However, prevention or early detection and treatment of potential adverse effects of immunosuppression is the only acceptable posttransplant strategy. An adequate patient compliance and close long-term follow-up have to be guaranteed. Moreover, as the procedure is elective and ischemia time is the shortest living donation seems most advantageous. Against this background willing healthy individuals (not requiring surgery for parathyroid disease) might also be considered as donors. Although identification and removal of parathyroid tissue from a normocalcemic healthy donor may be challenging due to small size of the parathyroid glands, it is generally considered as a safe procedure. Importantly, in the light of improved preoperative localization of parathyroid glands by innovative diagnostic tools (Technetium-sestamibi scintigraphy, contrast-enhanced ultrasonography) focused minimally invasive organ retrieval causing only minimal trauma and excellent cosmetic results has become standard.

## Conclusion

Parathyroid gland allotransplantation is a valuable and potentially definitive therapeutic option for patients with intractable persistent hypoparathyroidism. Provided that the well informed patient is willing to undergo transplantation despite of potential adverse effects of immunosuppressive therapy parathyroid allotransplantation can even be justified in nontransplant recipients. Living donation from healthy donors is a safe procedure that can be performed in minimally invasive technique.

## Abbreviations

CEUS, contrast-enhanced ultrasonography; FDA, food and drug administration; i.v., intravenous; MHC, Major histocompatibility complex; PTH, parathyroid hormone.
